# Therapeutic Hypothermia for Neonatal Hypoxic–Ischemic Encephalopathy – Where to from Here?

**DOI:** 10.3389/fneur.2015.00198

**Published:** 2015-09-14

**Authors:** Joanne O. Davidson, Guido Wassink, Lotte G. van den Heuij, Laura Bennet, Alistair J. Gunn

**Affiliations:** ^1^The Department of Physiology, The University of Auckland, Auckland, New Zealand

**Keywords:** hypothermia, ischemia, hypoxia–ischemia, neonate, neuroprotection, brain

## Abstract

Hypoxia–ischemia before or around the time of birth occurs in approximately 2/1000 live births and is associated with a high risk of death or lifelong disability. Therapeutic hypothermia is now well established as standard treatment for infants with moderate to severe hypoxic–ischemic encephalopathy but is only partially effective. There is compelling preclinical and clinical evidence that hypothermia is most protective when it is started as early as possible after hypoxia–ischemia. Further improvements in outcome from therapeutic hypothermia are very likely to arise from strategies to reduce the delay before starting treatment of affected infants. In this review, we examine evidence that current protocols are reasonably close to the optimal depth and duration of cooling, but that the optimal rate of rewarming after hypothermia is unclear. The potential for combination treatments to augment hypothermic neuroprotection has considerable promise, particularly with endogenous targets such as melatonin and erythropoietin, and noble gases such as xenon. We dissect the critical importance of preclinical studies using realistic delays in treatment and clinically relevant cooling protocols when examining combination treatment, and that for many strategies overlapping mechanisms of action can substantially attenuate any effects.

## Introduction

There is now compelling clinical evidence that mild induced hypothermia significantly improves survival and disability, including cerebral palsy and neurocognitive outcomes, in full-term infants with moderate to severe hypoxic–ischemic encephalopathy (HIE) (1), which persists into middle childhood (2, 3). The development of therapeutic hypothermia is a leading example of how sound physiological understanding combined with robust large animal models can support the development of effective clinical treatments. The use of highly translatable animal research should be at the forefront of our efforts to optimize hypothermia protocols, test potential combination therapies and ensure the safety and efficacy of potential treatments before human clinical trials.

Current hypothermia protocols have consistently involved starting treatment within the first 6 h of life, with systemic cooling to either 34.5 ± 0.5°C for head cooling, or 33.5 ± 0.5°C for whole-body cooling and continuing treatment for 48–72 h, as recently reviewed (1). These protocols significantly improve outcomes, but are only partially effective, with a number needed to treat of eight (1). That is to say, many infants still suffer severe brain damage and disability, even when treated with hypothermia. New ways to further reduce the burden of injury are still needed.

## The Evolution of Injury

**Figure 1:**
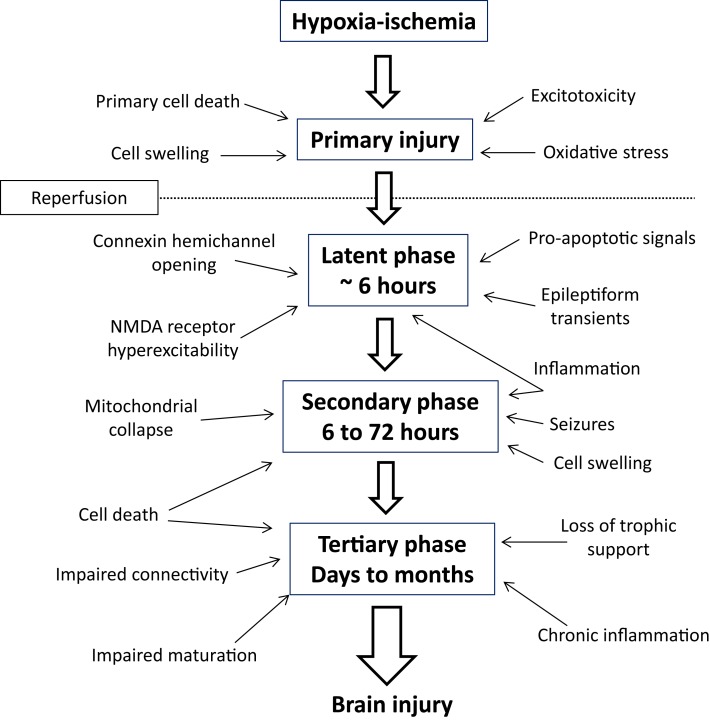
**Mechanisms of evolving neural injury in the primary phase, latent phase, secondary phase, and tertiary phase that contribute to long-term brain damage and disability**.

The critical observation from experimental studies *in vivo* and *in vitro*, and clinical observations that enabled the development of therapeutic hypothermia, is that HIE is not a single “event” but rather an evolving process leading to delayed cell death (Figure 1). During the immediate period of HI (the “primary” phase of the injury), high energy metabolites are depleted, leading to progressive depolarization of cells, severe cytotoxic edema (cell swelling) (4), and extracellular accumulation of excitatory amino acids due to failure of reuptake by astrocytes and excessive depolarization mediated release (5). Although neurons may die during a sufficiently prolonged period of ischemia or asphyxia, many neurons initially recover, at least partially, from the insult in a so called “latent” phase, only to develop progressive dysfunction and die many hours, or even days later. Magnetic resonance spectroscopy showed that many infants with evidence of moderate to severe asphyxia have initial, transient recovery of cerebral oxidative metabolism after birth, followed by secondary deterioration with cerebral energy failure from 6 to 15 h after birth (6). The severity of the secondary deterioration was closely correlated with neurodevelopmental outcome at 1 and 4 years of age (7), and infants with encephalopathy who did not show initial recovery of cerebral oxidative metabolism had extremely poor outcomes (6). An identical pattern of initial recovery of cerebral oxidative metabolism followed by delayed (“secondary”) energy failure is also seen after HI in the piglet, rat, and fetal sheep, and is closely correlated to the severity of neuronal injury (8–10). The timing of energy failure after HI is tightly coupled with the appearance of histologic brain damage (11), implying that it is primarily a function of evolving cell death.

After the bulk cell death during the secondary phase, there is a tertiary phase of repair and reorganization. During this period, new cell development and “rewiring” of surviving neuronal circuits is stimulated. At the same time, there is evidence that in some settings physiological apoptosis may be upregulated, which can impair new cell production and survival, leading to ongoing cell loss over many months (12–17). The precise mechanisms for this prolonged injury are not wholly clear, but may reflect in part persistent inflammation and epigenetic changes (18).

These concepts, that an acute, global insult can trigger evolving injury and that characteristic events are seen at different times after the insult, are central to understanding the causes of neonatal encephalopathy and how treatments, such as hypothermia, attenuate the evolution of injury.

## The Pharmacodynamics of Therapeutic Hypothermia

### When to cool

The partial protection with current hypothermia protocols found in clinical studies is likely at least in part related to the formidable clinical difficulties involved in starting hypothermia within the optimal window of opportunity (19). Preclinical studies in the near-term fetal sheep have shown that when hypothermia is started within the latent phase, at 90 min or 3 h after the end of ischemia, neuronal and oligodendrocyte cell death is dramatically reduced and brain activity recovers to near baseline levels (4, 20). In marked contrast, when treatment was delayed until late in the latent phase, 5.5 h after ischemia, only partial improvement in neuronal survival and recovery of EEG power was seen, with no improvement in oligodendrocyte survival (21, 22). When hypothermia was delayed until 8.5 h after the end of ischemia, after the onset of seizure activity in this paradigm, hypothermia was no longer associated with an improvement in cell survival or recovery of EEG activity (23). It is clear from these preclinical studies that hypothermia must be started during the latent phase, ideally within the first 3 h after ischemia, to achieve the best possible neuroprotective effect. This is consistent with clinical data from a recent cohort study that suggested that asphyxiated infants who were able to be cooled within 3 h of birth had better motor outcomes than when hypothermia was started between 3 and 6 h (24). However, in a typical randomized, controlled trial, hypothermia was only able to be started in 12% of infants within 4 h of birth (25).

Many infants with encephalopathy will have been exposed to HI well before birth and therefore there is an unavoidable, and often unknown, delay before clinical diagnosis. Characteristic features of neonatal encephalopathy include depression of the level of consciousness, respiratory depression, abnormal muscle tone and power, disturbance of cranial nerve function, and delayed seizures (26). However, as described above, the onset of seizures is associated with the phase of secondary deterioration, with a corresponding reduction in the efficacy of hypothermia (23). Thus, the most effective known way to improve outcomes after therapeutic hypothermia is through early diagnosis and initiation of treatment (27).

Given that many infants are born in small hospitals without neonatal intensive care units or access to therapeutic hypothermia, the neonatal transport team plays a key role in establishing and maintaining therapeutic hypothermia during transport to larger treatment centers. Establishing the optimal protocol for treating infants with hypothermia during transport is an ongoing area of research. Options include passive cooling by stopping active warming, and active cooling with ice packs or a servo-controlled blanket, as recently reviewed (28). For example, in a recent clinical trial, active cooling to a target temperature of 33.5°C, with a blanket servo-controlled to rectal or esophageal temperature probes, was associated with significantly more infants reaching target temperature during transport than during passive cooling (29). Moreover, active cooling reduced the average time to target temperature by approximately an hour. Further studies are important to determine whether this strategy can also improve neurological outcomes.

### Duration and depth of treatment

Preclinical studies suggest that to some extent the loss of efficacy associated with delayed onset of hypothermia can be salvaged by more prolonged cooling. For example, in adult gerbils, 12 h of hypothermia initiated 1 h after global ischemia effectively reduced hippocampal injury after 3, but not 5 min, of global ischemia (30). However, if the duration of hypothermia was extended to 24 h, near total preservation of CA1 neurons was achieved after 5 min of global ischemia (30). In adult rats, systemic hypothermia induced for either 12 and 24 h or 48 h (plus rewarming at a rate of 1°C/h) started 1 h after middle cerebral artery occlusion was associated with a significant reduction in neurological deficits with all treatment durations, however, motor deficits were only improved after cooling for 24 or 48 h (31). Similarly, selective brain cooling for 48 h plus rewarming at a rate of 1°C/h significantly reduced injury and behavioral impairment, whereas cooling for 12 h did not (32). These studies suggest that there is a complex relationship between the severity of insult, delay in treatment, duration of hypothermia, and method of inducing hypothermia, which all contribute to the effectiveness of treatment.

A recent study in the near-term fetal sheep found that extending the duration of delayed cerebral cooling, starting 3 h after cerebral ischemia, from 3 days until 5 days was not associated with any additional improvement in the recovery of EEG power or spectral edge frequency. Indeed, there was an apparent small but significant reduction in neuronal survival in the cortex and dentate gyrus (20). By contrast, in adult rats 2, 4, or 7 days of hypothermia had similar effects on neuronal survival in the CA1 of the hippocampus and more prolonged cooling was not associated with any adverse effects on markers of brain plasticity (33). Furthermore, 21 days of mild focal hypothermia was not associated with any adverse effects on behavior or cell death in healthy adult rats (34). Although it is reassuring that even such a prolonged period of hypothermia did not have adverse effects in the healthy brain, it is important to reflect that we cannot necessarily assume that extended durations of hypothermia will not impair recovery from HI.

The depth of cooling is also important. In near-term fetal sheep, brain cooling has been shown to be associated with a steep, sigmoidal relationship between brain temperature and protection, with neuroprotection achieved below a brain temperature of 34°C but no further improvement with greater cooling (4). Similarly, in term piglets, whole-body cooling with a 3.5°C reduction in core temperature (from control values of 38.5 to 35°C) prevented secondary failure of oxidative metabolism and reduced neuronal cell death (35). Broadly similar improvements in neuronal loss were achieved with a reduction in core temperature by 3.5 or 5°C from 2 to 26 h after HI (36). Of concern, deeper cooling to 8.5°C below control values was associated with markedly reduced neuroprotection (36), and increased risk of hypotension and cardiac arrest (37).

Consistent with this preclinical evidence, a large randomized controlled trial of 72 h of hypothermia to 33.5°C compared with either prolonged hypothermia for 120 h or deeper cooling to 32°C, was stopped early because longer duration, lower temperature and the combination of longer duration and lower temperature were associated with a trend toward a higher risk of death in the neonatal period (38). It will be of considerable interest to know whether these interventions were associated with any effect on neurological outcomes in surviving infants, even though the trial was stopped half way because it was considered improbable that there could be a net effect on death or disability.

### Rate of rewarming

The published clinical trials of therapeutic hypothermia have consistently aimed to rewarm neonates after hypothermia at a rate of 0.5°C/h (39, 40). However, it is notable that this is not based on strong evidence. In near-term fetal sheep, rapid, spontaneous rewarming over approximately 30 min, after 72 h of head cooling, was associated with increased electrographic seizures in 5/9 animals in the ischemia–hypothermia group compared to 1/13 animals in the ischemia–normothermia group (41). However, the absolute effect was modest and it is notable that this protocol markedly improved EEG recovery and neuronal survival despite these transient EEG changes (4). In neonatal piglets, rapid rewarming (4°C/h) after post-HI hypothermia was associated with increased cortical apoptosis compared with slow rewarming (0.5°C/h) (42). However, the initial period of hypothermia was only 18 h. Thus, it is not clear from this study whether it was slower rewarming *per se* that improved outcome or whether it was the extended duration of mild hypothermia compared to the rapidly rewarmed group.

In adult rats, speed of rewarming had no effect on biochemical or behavioral recovery after 20 min of hypothermia, but rapid rewarming was associated with a greater earlier change in cardiac output and heart rate in the rapidly rewarmed group compared to the slowly rewarmed group (43). Similarly, in adult gerbils, rapid rewarming over 30 min after 2 h of hypothermia was associated with transient uncoupling of cerebral circulation and metabolism, with a transient increase in extracellular glutamate and lactate (44). Furthermore, after traumatic brain injury in adult rats rapid rewarming over 15 min, after 1 h of hypothermia, exacerbated traumatic axonal injury and impaired cerebrovascular responsiveness compared to rewarming over 90 min (45, 46). However, these studies examined rewarming after very short, subtherapeutic periods of hypothermia.

These data suggest that rapid rewarming after short intervals of hypothermia can have undesirable physiologic and neuronal effects but it is not yet known how the speed of rewarming affects the development of injury and long-term outcome. Further investigation, using clinically established hypothermia protocols are needed to establish whether the speed of rewarming after HI is important or not for optimal neuroprotection.

## Can Combination Treatment Augment Hypothermic Neuroprotection?

Given the evidence discussed above that current clinical hypothermia protocols are reasonably close to optimal (27), the other key strategy to improve neonatal neuroprotection would be to combine known effective hypothermia protocols with other putative neuroprotective agents. Given that mild hypothermia is now standard of care, it is essential to test potential treatments in combination with hypothermia. This is particularly important as overlap with the wide array of reported mechanisms of action of hypothermia (47) may attenuate any additive effects, as discussed below (48–50). There are of course very large numbers of potential strategies; in this review we examine some leading examples, as summarized in Table 1.

**Table 1:** **Summary of the evidence for additive neuroprotective effects with hypothermia and potential combination treatments**.

Combination treatment	Species	Age	Additive effects	Hypothermia started	Other intervention started	Comment
**Anti-inflammatory/neuroregenerative**						
Erythropoietin	Non-human primates (51)	Full term	Yes (survival, motor, cognitive responses, cerebellar growth, and MRI)	Immediately	30 min	Hypothermia for 72 h
	Neonatal rat (52)	P7	No (sensorimotor, histopathology)	1 h	Immediately	Hypothermia for 8 h
	Neonatal rat (53)	P7	Borderline (sensorimotor, histopathology)	Immediately	Immediately	Hypothermia for 3 h
Stem cells	Neonatal rat (54)	P7	Yes (histology, MRI, functional)	6 h	6 h	Hypothermia: 32°C for 24 h
**Anti-oxidative/anti-inflammatory**						
Melatonin	Newborn piglet (55)	Full-term	Yes (MRS, histology)	2 h	10 min	Hypothermia for 26 h
**Anti-apoptotic**						
IGF-I	Fetal sheep (49, 56)	0.85 gestation (term equivalent)	No (EEG and histology)	5.5 h	4.5 h	Hypothermia for 72 h
**Anticonvulsant agents**						
Xenon	Neonatal rats (57)	P7	Yes (histology, functional)	4 h	4 h	Hypothermia for 90 min
	Newborn piglet (58)	Term	Yes (neuropathology, clinical neurology)	<40 min	30 min	Hypothermia for 12–24 h
	Newborn piglet (59)	Term	No (trend, MRS, histology)	2 h	2 h	Hypothermia for 24 h
	Humans (60)	Term	Yes (seizures only)	<12 h	<12 h	Reduced seizures
Phenobarbital	Neonatal rats (61)	P7	Yes (histology, MRI, functional)	1–3 h	15 min	Hypothermia: 30°C for 3 h
Dizocilpine	Fetal sheep (50)	0.7 gestation	No (EEG and histology)	5.5 h	15 min	Hypothermia for 72 h
**Connexinhemichannel blockade**						
Cx43 mimetic proteins	Fetal sheep (48)	0.85 gestation (term equivalent)	No (EEG and histology)	3 h	3 h	Hypothermia for 72 h

*Cx43, connexin 43; EEG, electroencephalography; IGF-I, insulin-like growth factor I; MRI, magnetic resonance imaging; MRS, magnetic resonance spectroscopy; functional, neurobehavioral tests*.

### Erythropoietin

Erythropoietin (EPO) has a central role in erythropoiesis and is now routinely used as a treatment for anemia in the premature infant (62). In addition, there is increasing clinical and experimental evidence that recombinant human EPO (rhEPO) may be neuroprotective after HIE, by binding to the EPO receptor (EPOR) on neurons and glia. In both the adult and neonatal brain, EPO can promote expression of anti-apoptotic relative to pro-apoptotic genes, inhibit caspase activation, attenuate oxygen free radicals, and the inflammatory response to HI, and increase neurogenesis (63). rhEPO treatment after HI and stroke in normothermic neonatal rodents improved recovery of sensorimotor function, and behavioral and cognitive performance and histological integrity (63).

Moreover, there is reasonable clinical evidence of the safety of rhEPO. A recent meta-analysis of five studies involving 233 patients, including very low birth weight infants and premature infants, suggested that rhEPO administration improved neurodevelopmental outcome and was not associated with adverse effects (64). In full-term neonates with HIE, two studies have also shown that rhEPO treatment was safe. Low-dose rhEPO (300 or 500 U/kg) was associated with a reduced risk of death or disability in term infants with moderate, but not severe, HIE (65). Furthermore, high-dose rhEPO (2,500 U/kg) started within the first 48 h of life improved neurodevelopmental outcome in term neonates with mild/moderate HIE and was also associated with a significant reduction in seizure activity, improved abnormal EEG background at 2 weeks, and decreased neurologic abnormalities at 6 months (66).

Despite this very encouraging evidence for safety and independent neuroprotection, there is relatively limited evidence that giving EPO can augment hypothermic neuroprotection. Moreover, it is plausible that hypothermia may reduce metabolism of rhEPO or attenuate activation of intracellular pathways triggered by EPO. After HI in P7 neonatal rats, investigators have variably reported both no significant neuroprotection with combined EPO with hypothermia (52), and a borderline additive effect of rhEPO plus hypothermia on sensory-motor function but not on brain histology (53). More encouragingly, in non-human primates after umbilical cord occlusion immediately before birth, early treatment with hypothermia and EPO improved motor and cognitive responses, cerebellar growth, and reduced death or disability (51). However, it is important to note that treatment was begun much earlier than has been possible in any human randomized controlled trials of neuroprotection, with EPO given 30 min after birth, and passive cooling begun immediately after birth, followed by active cooling after resuscitation, and always before 3 h after birth.

Two RCT phase I/II trials investigating combined cooling with rhEPO in infants with HIE (the DANCE and NEATO trials, NCT01471015/NCT01913340) are listed on ClinicalTrials.gov. These ongoing studies are examining safety, pharmacokinetics, and efficacy of combined treatment.

### Melatonin

Melatonin (*N*-acetyl-5-methoxytryptamine) is a naturally occurring indolamine secreted by the pineal gland to regulate circadian rhythm that also has anti-oxidant properties (63). It has clinical potential as a prophylactic treatment for fetuses at high risk of perinatal hypoxia as it readily crosses the placenta (63). When given before or immediately after HI, melatonin is neuroprotective in postnatal rodents, as previously reviewed (63). In term-equivalent fetal sheep, maternal prophylactic melatonin (1 mg total) given before 10 min umbilical cord occlusion was associated with reduced brain lipid peroxidation, neuronal death, microglial activation, and astrogliosis (67). In preterm fetal sheep at 0.7 gestation, maternal low-dose melatonin infusion was associated with faster fetal EEG recovery, delayed onset of seizures, improved survival of mature oligodendrocytes, and reduced microglial activation in the periventricular white matter (68).

There is emerging evidence for neuroprotection with post-insult treatment with melatonin. In preterm fetal sheep at 0.6 gestation, fetal infusion of high-dose (20 mg/kg) melatonin for 6 h from shortly after umbilical cord occlusion was associated with reduced apoptosis and microglia in the white matter, although cell survival was not quantified (69). High-dose melatonin (5 mg/kg/h over 6 h) given shortly after HI in postnatal term piglets strikingly augmented protection from subsequent therapeutic hypothermia on both MR spectroscopy markers of anaerobic stress, and histopathology (55). However, melatonin was given 10 min after asphyxia, which is not clinically realistic, whereas the start of hypothermia was delayed until 2 h and was only continued until 26 h. Thus, again this is much earlier initiation of treatment than has been achieved in human randomized controlled trials to date.

An important potential limitation of these studies is that melatonin is a hydrophobic molecule and therefore ethanol is frequently used as a diluent, which may adversely affect the developing brain. Postnatally, in term piglets, very high-dose melatonin (10 mg/kg) dissolved in ethanol was associated with hypotension and increased inotrope requirements after HI (55); it is unknown whether the melatonin or ethanol or the combination mediated this adverse effect. Furthermore, in a recent study of prophylactic maternal melatonin before severe asphyxia in preterm fetal sheep (68), there was evidence that although melatonin was associated with faster recovery of the fetal EEG and improved white matter recovery compared to the 2% ethanol vehicle, both melatonin and ethanol vehicle were associated with similar overall improvement in neuronal survival in the striatum and reduced post-asphyxial seizures (68). By contrast, ethanol was also associated with greater neuronal loss in the CA3 and CA4 regions of the hippocampus and reduced white matter proliferation, with greater induction of amoeboid microglia. These findings strongly suggest that even small amounts of ethanol may partly confound the neuroprotective effects of melatonin and, thus, that it is essential to test alternate diluents.

### “Stem” cells

Over the past decade, there has been increasing interest in the use of stem or progenitor cells to improve recovery from neonatal HIE and even for older children with established cerebral palsy. The media has reported a handful of cases showing apparent recovery, leading to inflated expectations and pressure from parents of children with cerebral palsy to use stem cells as a routine treatment strategy, despite a lack of rigorous evidence (70). In preclinical studies in rat and mouse models, the effects of a wide range of different stem/progenitor cell preparations given after neonatal HIE have been highly variable, as reviewed (70), likely reflecting considerable differences in type, dose, and timing of infusion of the stem cells after injury.

There is increasing evidence that functional improvements can occur without significant functional engraftment (70), suggesting that the effects of stem/progenitor cells are likely mediated through neurotrophic and immunomodulatory mechanisms. Consistent with this, in rabbits exposed to intrauterine HI at 70% gestation, subsequent infusion of human umbilical cord blood cells at birth was associated with a dose-dependent improvement in the motor problems despite little penetration of the stem cells into the brain (71). Promisingly, combined treatment with mesenchymal stem cell therapy and 24 h of hypothermia, started 6 h after the end of HI in the P7 rat, showed greater improvement with combined treatment than either treatment alone (54), as measured at P42 by MRI and functional behavioral tests.

There are currently 12 clinical trials of stem cell therapy for neonatal HI or cerebral palsy listed on Clinicaltrials.gov as recruiting, underway or completed. One small double-blind randomized, placebo-controlled trial in 96 children with cerebral palsy receiving rehabilitation therapy found that treatment with a combination of umbilical cord blood and rhEPO ameliorated motor and cognitive dysfunction after 6 months of treatment more than rehabilitation therapy with or without rhEPO (72). Thus, stem cell therapy shows promise as a treatment for HIE, whether given alone or in combination with therapeutic hypothermia (71). In order to optimize the potential of this treatment, systematic preclinical studies of the mechanisms of action, optimal dosing and timing, and type of stem cells are now needed.

### The noble gases: Xenon

Xenon is an inert noble gas used for its anesthetic properties, mediated via competitive binding at the glycine binding site of the *N*-methyl-d-aspartate glutamate receptor (73). In addition to potentially attenuating excitotoxicity, xenon may also activate pro-survival kinases, such as p-Akt and the anti-apoptotic factor Bcl-2, and potentially inhibit opening of the mitochondrial permeability pore (74).

There is evidence of additive protection from combined xenon and hypothermia treatment. Xenon and hypothermia administered together either immediately or as late as 4 h after HI in neonatal rats significantly reduced apoptotic cell death and loss of brain matter while improving long-term neurological motor function and coordination (57, 63). In the newborn piglet, the combination of xenon with whole-body cooling was associated with a 75% reduction in global neuropathology after perinatal asphyxia (58). However, hypothermia was only continued for either 12 or 24 h in this study. In a similar paradigm, xenon-augmented hypothermia reduced cerebral MRS abnormalities and cell death markers in some brain regions compared with no treatment, although the effect was not significant compared to hypothermia alone (59). In a small clinical study, 5/14 full-term neonates with HIE treated with 72 h of hypothermia developed seizures, which was suppressed during xenon ventilation, recurred on withdrawal of xenon and then again suppressed on reintroduction of xenon (60).

The limited natural availability of xenon, and thus high price, means that it needs to be used with a recirculating ventilator (75), and thus even if it is effective, it is unlikely to ever be available outside of tertiary units. The feasibility of combined treatment with xenon and hypothermia is being evaluated in phase 2 trials (TOBYXeNCT00934700 and CoolXenon2-NCT01545271) (74). Encouragingly, in P7 rat pups, the relatively inexpensive noble gas, Argon, provided highly comparable neuroprotection after HI to xenon (76).

### Anticonvulsants

Although seizures in infants suffering HIE are associated with adverse outcomes (77, 78), it remains very unclear whether these seizures are the cause of injury or simply reflect the evolution of pre-existing injury. Thus, it is unknown whether blocking seizure activity reduces the development of brain injury (79). Mild hypothermia does seem to reduce the overall burden of seizures after moderate HIE (80), but they remain common during cooling and highly associated with adverse outcomes (81). There is considerable interest as to whether anticonvulsant therapy can augment hypothermic neuroprotection. In neonatal rats, phenobarbital treatment from 15 min after HI in combination with hypothermia started either 1 or 3 h after HI was associated with a significant improvement in sensorimotor performance and reduced brain damage (61). However, hypothermia was only administered for 3 h and was markedly delayed compared to injection of phenobarbital.

In near-term fetal sheep, infusion of the *N*-methyl-d-aspartate receptor antagonist, dizocilpine, 6 h after the end of HI, completely suppressed seizure activity, but only reduced neuronal cell death in the less susceptible lateral cortex (temporal lobe) and hippocampus, but not in the highly susceptible parasagittal cortex (82). Potentially, this may indicate that to achieve neuroprotection, treatment with anticonvulsants would need to be initiated *before* seizures start. Consistent with this concept, in preterm fetal sheep, dizocilpine infusion started shortly after severe asphyxia was associated with selective neuroprotection of the striatum (83), but neuroprotection was not additive with delayed mild hypothermia (50). These findings suggest that hypothermia may in part be acting by suppressing neural injury related to excessive glutamatergic activity.

Clinically, in a retrospective study of infants administered phenobarbital before treatment with hypothermia for HIE, combined treatment did not improve the composite outcome of neonatal death or the presence of an abnormal post-treatment brain MRI (84). Thus, at present this strategy requires further robust preclinical testing before formal controlled trials can be considered.

### Anti-apoptotic factors: Insulin-like growth factor-1

Insulin-like growth factor-1 (IGF-I) is one of the large array of growth factors that contributes to regulating brain growth. IGF-I is potently anti-apoptotic, as well as promoting neural stem cell proliferation, differentiation, maturation, myelination, neurite outgrowth, and synaptogenesis (85). There is consistent evidence that post-ischemic administration of exogenous IGF-I can attenuate the severe delayed, post-ischemic neuronal and oligodendrocyte cell loss and associated demyelination after HI in the rat and cerebral ischemia in near-term fetal sheep (85). For example, in term-equivalent fetal sheep, IGF-I given as a 1 h intracerebroventricular infusion 90 min after cerebral ischemia was associated with reduced loss of oligodendrocytes in the intragyral white matter, reduced demyelination, reduced tissue swelling, but upregulation of astrocytes and microglia (56). By contrast, delayed co-treatment with IGF-I started 4.5 h after ischemia plus mild hypothermia in the near-term fetal sheep did not improve white matter damage or reduce caspase-3 activation compared to hypothermia alone. This suggests that their mechanisms of neuroprotection are overlapping, likely through the anti-apoptotic effects of hypothermia (49, 86).

### Blockade of connexin hemichannels

One of the most striking features of HI brain injury is that injury consistently spreads over time from severely affected regions to areas that were originally intact (87). This pattern is consistent with the long-standing hypothesis that cell to cell communication might contribute to spreading injury. The gap junctions that link adjacent cells to allow transport of small molecules, ions and second messengers (88), are formed by docking of hexamer hemichannels (connexons) from the adjacent cells. There is increasing evidence that these connexin hemichannels are not just passively waiting to dock, but are themselves active under normal physiological conditions, for example, through purinergic signaling by regulated release of ATP (88). Critically, pathological conditions such as ischemia may cause unregulated opening, compromising the resting membrane potential, and allowing transmitters such ATP or glutamate to be released into the extracellular space (88).

An elegant study from Orellana et al. showed that Connexin43 hemichannels can open *after* hypoxia in cultured astrocytes (89), as shown by increased dye uptake in Connexin43-containing astrocytes, but not Connexin43-deficient astrocytes, and that blockers of Connexin43 hemichannels prevented dye uptake and death of astrocytes. In fetal sheep, intracerebroventricular infusion of a mimetic peptide at a dose concentration that blocks Connexin43 hemichannels (90) started 90 min after either cerebral ischemia or profound asphyxia and continued for 25 h improved EEG recovery and reduced white and gray matter damage (91, 92). In the term-equivalent fetal sheep, this mimetic peptide infusion was associated with striking reduction in status epilepticus after ischemia, consistent with the hypothesis that connexin hemichannels play a key role in propagating these intense seizures (91).

By contrast, when the start of connexin hemichannel blockade was delayed until 3 h after ischemia, there was no improvement in cell survival or recovery of brain activity, despite attenuated seizure activity and secondary cell swelling (48). When connexin hemichannel blockade was combined with hypothermia from 3 h after ischemia, no additive neuroprotective effects were seen, again suggesting that the mechanisms of action of hypothermia likely overlap with those of connexin hemichannel blockade.

## Conclusion

Therapeutic hypothermia is now well established as standard care for infants with moderate to severe HIE. Recent preclinical studies and a large clinical randomized trial suggest that current treatment protocols are reasonably close to optimal. Further improvements in outcome are highly likely to arise from improved identification of affected infants that would allow earlier initiation of treatment after resuscitation. An important remaining pragmatic question is whether slower rewarming after therapeutic hypothermia may improve outcomes. Further research to systematically test proposed new neuroprotective treatments with hypothermia is now critical. An important limitation is that many potential interventions appear to work through mechanisms of action that overlap with hypothermia. Thus, it will be important to target strategies that act through complimentary mechanisms to hypothermia. It is very encouraging that both stem cells and rhEPO have actions that extend into the tertiary phase, with known effects on restoration, migration, maturation, and trophic support (63, 70). This strongly suggests that these are promising candidates for treatment in combination with, or even after, early mild cooling.

Finally, effective translation will require that the effects of such therapeutic strategies are characterized in translatable large animal models, using clinically realistic administration protocols, including a realistic delay in treatment, and tested in combination with clinically established hypothermia protocols that are not impaired by overly short duration of treatment.

## Author Contributions

All authors contributed to drafting the manuscript and approved the final version.

## Conflict of Interest Statement

The authors declare that the research was conducted in the absence of any commercial or financial relationships that could be construed as a potential conflict of interest.

## Funding

The authors work in this review was supported by grants from the Health Research Council of New Zealand (grant number 12/613 and 14/216), the Auckland Medical Research Foundation (grant number 1108004) and the Lotteries Board of New Zealand (grants number 209214 and 340855).
